# The association between non-depolarizing neuromuscular blockade agents and survival to discharge in dogs undergoing mechanical ventilation: a multi-center retrospective study of 227 dogs (2010–2020)

**DOI:** 10.3389/fvets.2025.1539138

**Published:** 2025-03-05

**Authors:** Lena Ngo, Rebecca Walton, Jacob Wolf, Nyssa Levy, Tasia Ludwik, Britt Thevelein, April Blong, Jiazhang Cai, Jonathan Mochel

**Affiliations:** ^1^Department of Critical Care, VCA West Los Angeles, Los Angeles, CA, United States; ^2^Department of Veterinary Clinical Sciences, Iowa State University, Ames, IA, United States; ^3^Department of Small Animal Clinical Practice, University of Florida, Gainesville, FL, United States; ^4^Department of Small Animal Clinical Sciences, Michigan State University, East Lansing, MI, United States; ^5^Department of Veterinary Clinical Sciences, University of Minnesota, St. Paul, MN, United States; ^6^College of Veterinary Medicine, University of Georgia, Athens, GA, United States; ^7^Precision One Health Initiative, The University of Georgia, Athens, GA, United States

**Keywords:** acute respiratory distress, hypoxemia, hypoventilation, neuromuscular blockade, ventilator complications, ventilator discontinuation

## Abstract

**Objective:**

To evaluate the association between neuromuscular blockade agent (NMBA) use and outcome in dogs undergoing mechanical ventilation (MV), including survival to discharge, and complications.

**Methods:**

The medical records for 227 mechanically ventilated dogs were reviewed for NMBA use, parameters of respiratory status (PaO_2_, PCO_2_, PF ratio, SpO_2_), MV settings, MV complications, and survival outcome.

**Results:**

The NMBA and non-NMBA groups included 28 and 199 dogs, respectively. The median partial pressures of oxygen in arterial blood (PaO_2_) in the NMBA and non-NMBA groups were 63 and 57 mmHg, respectively (*P* = 0.24). The median partial pressures of blood carbon dioxide levels were 58 and 51 mmHg, respectively (*P* = 0.07). The pulse oximetry percentage (SpO_2_) prior to initiation of MV were 88 and 94%, respectively (*P* = 0.02). The median PF ratios prior to MV were 90 and 215, respectively (*P* = 0.02). The median durations of MV were 18 and 24 h, respectively (*P* = 0.32). Eight (28.6%) dogs that received NMBAs survived to discharge, while 51 dogs (32.3%) that did not receive NMBAs survived to discharge (*P* = 0.87). Both PF ratio and SpO_2_ values were significantly lower in dogs that received NMBAs compared to dogs that did not (*P* = 0.02 and *P* = 0.02, respectively). There was no significant difference in tidal volume or peak inspiratory pressure at the time of MV initiation (*P* = 0.17 and *P* = 0.09, respectively). There was no significant difference between the incidence of complications in dogs that received NMBAs and those that did not (*P* = 0.08).

**Conclusion:**

This study revealed no statistical significance between NMBA use and survival or complications. However, dogs in the NMBA group likely had more severe hypoxemia than the non-NMBA group, as indicated by their lower PF ratios and SPO_2_ values prior to initiation of mechanical ventilation. The similarities in survival rate between the NMBA and non-NMBA patient populations, despite higher severity of respiratory pathology in the NMBA group, may suggest a potential therapeutic benefit to NMBA use for MV patients. Further investigation into the use of NMBAs in patients undergoing MV are warranted.

## 1 Introduction

Mechanical ventilation (MV) may be utilized in the management of critically ill patients suffering from respiratory failure, circulatory collapse, and/or an impaired state of consciousness (1). Protocols for MV vary extensively regarding ventilatory strategies and pharmacologic intervention. While standard treatment strategies, such as lung-protective ventilation, have consistently improved patient outcome, the benefits of using neuromuscular blockade agents (NMBAs) have been widely debated (2). Neuromuscular blockade agents mimic the structural characteristics of acetylcholine, which prevent depolarization of the muscle fiber and subsequently produce a state of generalized muscular paralysis (3). The resultant respiratory muscle paralysis may provide therapeutic benefits in MV including decreased oxygen consumption, prevention of patient-ventilator dyssynchrony, and reduced risk of barotraumatic complications (4–6). In people undergoing MV for severe acute respiratory distress syndrome (ARDS), early administration of a NMBA improved the adjusted 90-day survival (7). While the benefits of NMBAs have been evaluated in humans, no literature regarding the use of NMBAs exists in veterinary medicine.

The goals of this study were to evaluate the association between NMBA use and outcome, including survival to discharge. Secondary objectives included the evaluation of NMBA use and complications noted during MV. We hypothesized that the use of NMBAs would be associated with a higher survival to discharge, and a lower complication rate.

## 2 Materials and methods

### 2.1 Case selection

The computerized medical record systems at five university teaching hospitals were searched for dogs undergoing MV from January 2010 to December 2020. Search criteria included financial and medical record codes for “mechanical ventilation.” Dogs were included in data analysis if they underwent long-term MV, which was defined as MV for reasons other than general anesthesia, for any reason. Dogs were excluded from data collection and analysis if medical records were incomplete. The following data was collected on spreadsheet based on retrospective evaluation of records: signalment, disease process indication for MV, duration of MV, use of lung protective strategy, peak inspiratory pressures, tidal volume and PEEP at the initiation of MV, NMBA use, complications, and survival to discharge. Lung protective strategy was defined as restrictive tidal volume and use of PEEP. Additionally, results from pulse oximetry and venous or arterial blood gas were recorded when available. Complications evaluated from the records included: pneumothorax, ventilator associated pneumonia, development of multi-organ dysfunction, ocular ulcerations, or any other suspected complications including new onset seizures or allergic reactions.

### 2.2 Statistical analysis

The primary goal of this study was to identify the relationships between study variables, including the primary indication for MV, the duration of MV, survival to discharge, and complications. The selection of variables were made a priori based on clinical relevance. These variables were mathematically categorized into binary, numerical, and multi-dimensional types. Binary variables, such as survival and complications, were converted to binary values (0 for no and 1 for yes). Numerical variables, including the length of hospitalization and the number of total weaning attempts, retained their original values. Multi-dimensional variables, such as the drugs used for induction and maintenance, were converted into multi-dimensional vectors, with each element representing a specific category as a binary value (0 or 1) indicating the presence or absence of that category. To analyze the relationships among these variables, various statistical tools were employed:

1. Comparison between two binary variables: a two-sample test for equality of proportions with continuity correction was used, and the results were visualized using pie charts.
2. Comparison between binary and numerical variables: differences in the distributions of numerical variables stratified by binary variables were tested using the Welch Two Sample *t*-test for normal distributions and the Wilcoxon rank sum test with continuity correction for non-normal distributions. Results were visualized using density plots.
3. Comparison between two numerical variables: The Pearson's product-moment correlation was calculated to test for significant correlations, with results visualized using correlation plots.
4. Comparison between binary and multi-dimensional variables: multi-dimensional variables were treated in a high-dimensional space, and differences were tested using the PERMANOVA test and permutation tests for variance. The PERMANOVA test assessed distribution differences, while permutation tests determined if differences were due to dispersion. Results were visualized using PCoA plots, with ellipses illustrating the dispersion and centroids of each distribution.
5. Comparison between two multi-dimensional variables and between numerical and multidimensional variables: Canonical Correlation Analysis (CCA) was used to test correlations among multi-dimensional variables. *P* < 0.05 were considered statistically significant. Statistical analyses were performed using R software version 4.3.1 [Wickham H (20). ggplot2: Elegant Graphics for Data Analysis. Springer-Verlag New York. ISBN 978-3-319-24277-4].

## 3 Results

A total of 239 dogs undergoing MV were identified through the medical records database. Twelve dogs were excluded due to incomplete medical records; therefore 227 dogs were included in the evaluation. Ninety dogs were castrated males, 85 dogs were spayed females, 29 were intact males and 23 were intact females. The median weight was 9.6 kg [interquartile range (IQR) 5.19–22.7 kg]. The median age was 7 years (IQR 2–10 years). The most common breeds represented were mixed breed dogs (32), Dachshund (19), Chihuahua (16), Yorkshire Terrier (14), Labrador Retriever (11), Pomeranian (7), Maltese (7) and French Bulldog (6). One hundred and thirty dogs (57%) underwent MV for hypoxemia, 85 dogs underwent MV for hypoventilation (38%) and 12 dogs (5%) underwent MV for a combination of hypoxemia and hypoventilation. Of the dogs treated due to hypoxemia, 35 were diagnosed with pneumonia, 29 were diagnosed with ARDS, 25 congestive heart failure (CHF), 24 had pulmonary contusions, 5 had other unknown pulmonary disease, 5 had pulmonary hemorrhage, 4 had non-cardiogenic pulmonary edema, 2 had pulmonary fibrosis, 1 dog had each of the following: submersion injury, neoplasia, pulmonary thromboembolism. Of the dogs undergoing MV for hypoventilation, 22 were diagnosed with intracranial disease, 18 with lower motor neuron disease, 15 with cervical myelopathies, 13 with post cardiac arrest syndrome, 12 with acute intoxication and 5 with upper airway obstruction.

A total of 28 dogs received NMBAs during MV, either at the initiation of MV or during maintenance of MV. Seven dogs received NMBAs for initiation of MV. All 28 dogs received intermittent boluses or a constant rate infusion of NMBAs during MV. All dogs received atracurium, no other NMBA was utilized. Fifteen dogs received NMBAs as a constant rate infusion, ranging from 0.04 to 0.1 mg/kg/h and 9 dogs received NMBA as intermittent bolus only, ranging from 0.1 to 0.2 mg/kg IV and 3 dogs received atracurium as a bolus, followed by a constant rate infusion. The exact reason for NMBA administration, including facilitation of sedation or management of dyssynchrony was not available. The median weight of dogs receiving NMBA was 6.15 kg (2.8–13.6 kg) and the median age was 3.5 years (IQR 1–12 years). Twenty-four dogs that received NMBAs (85.7%) underwent MV for hypoxemia while 4 dogs (14.3%) underwent MV for hypoventilation. At the time of MV initiation for dogs receiving NMBAs, partial pressure of oxygen in arterial blood (PaO_2_) was noted in 10 dogs (36%) with a median of 63 mmHg (range 52–241 mmHg) and pulse oximetry was available in 15 dogs with a median of 88% (range 76–97%). The PF ratio was available in 8 dogs with a median of 90 (IQR 58–482). Partial pressure of carbon dioxide levels, both venous and arterial, were available in 27 dogs with a median of 58 mmHg (IQR 28–130 mmHg). Twenty dogs (71%) of dogs were ventilated with lung protective ventilation strategies. Median tidal volume at initiation of MV was 8.5 mL/kg (range 4.7–13 mL/kg) and median peak inspiratory pressure at initiation of MV was 18 cmH_2_O (range 8–37 cmH_2_O). Median PEEP was 8 cmH_2_O (range 2–12 cmH_2_O). Duration of MV was a median of 18 hours in dogs receiving NMBAs (range 2–192 h). Eight dogs (28.6%) receiving NMBAs were successfully discontinued from MV, and all of those dogs survived to discharge. One dog (3.5%) receiving NMBAs developed a pneumothorax as a complication of MV. That dog was undergoing MV for non-cardiogenic pulmonary edema secondary to head trauma and this dog died naturally. No dogs were noted to develop new onset seizures or evidence of allergic reactions.

One-hundred and 98 dogs did not receive NMBAs. The median weight of dogs that did not receive NMBA was 10.65 kg (IQR 5.5–23 kg) and the median age was 7 years (IQR 2.25–10 years). The majority of dogs, 109 (54.4%), underwent MV for hypoxemia while 89 dogs (45.6%) underwent MV for hypoventilation. At the time of MV initiation, PaO_2_ was noted in 57 (29%) dogs with a median of 89.8 mmHg (range 20–498 mmHg) and pulse oximetry was available in 107 dogs with a median of 94% (IQR 83–100%). The PF ratio was available in 44 dogs with a median of 214.5 (range 33–590) Partial pressure of carbon dioxide levels were available in 124 dogs with a median of 51.3 mmHg (range 22–128 mmHg). One-hundred and 60 dogs (80%) were ventilated with lung protective strategies with a median tidal volume of 9 mL/kg (range 2–25 mL/kg) at the time of MV initiation and a median peak inspiratory pressure of 15 cmH_2_O (range 5.5–35 cmH_2_O). Median PEEP was 5 cmH_2_O (range 0–12 cmH_2_O). The median duration of MV was 24 h (range 1–210 h). Seventy-two dogs (36%) were successfully discontinued from MV, and 61 dogs (30.8%) survived to discharge. Forty-three dogs (21.7%) developed complications while on MV, 23 dogs developed ventilator-associated pneumonia, 14 dogs developed multiorgan dysfunction, and 6 dogs developed pneumothorax. No dogs were noted to develop new onset seizures or evidence of allergic reactions. Fourteen dogs that developed complications (32.6%) survived to discharge.

Dogs that received NMBAs were significantly more likely to be mechanically ventilated for hypoxemia, compared to hypoventilation (*P* = 0.004). There was no significant difference in the duration of mechanical ventilation or weaning success in dogs that received NMBAs compared to dogs that did not (*P* = 0.32 and *P* = 0.39, respectively; Table 1). There was no significant difference in survival to discharge between dogs that received NMBAs compared to dogs that did not (*P* = 0.87). Overall, 45 dogs (20%) experienced a complication and there was no significant difference between the incidence of complications in dogs that received NMBAs and those that did not (*P* = 0.08). Prior to the initiation of MV, both PF ratio and SpO_2_ values were significantly lower in dogs that received NMBAs compared to dogs that did not (*P* = 0.02 and *P* = 0.02, respectively). There was no significant difference in CO_2_ values prior to MV initiation (*P* = 0.07). The majority of dogs in both groups had lung protective ventilation strategies used. There was no significant difference in tidal volume or peak inspiratory pressure at the time of MV initiation between dogs that did receive NMBAs and those that did not (*P* = 0.17 and *P* = 0.09, respectively). Peak end expiratory pressure was significantly higher in the dogs that received NMBAs compared to those that did not (*P* < 0.01).

**Table 1:** In dogs undergoing mechanical ventilation, comparison between use of neuromuscular blocking agents (NMBA) compared to dogs that did not receive NMBA agents.

	**NMBA group (*n* = 28)**	**Control group (*n* = 198)**	***P*-Value**
Indication for MV			
Hypoxemia	24 (86%)	109 (55%)	
Hypoventilation	4 (14%)	89 (45%)	P = 0.004^*^
Survival to discharge	8 (28.6%)	61 (30.8%)	*P* = 0.87
Discontinuation of MV	8 (28.6%)	72 (36.4%)	*P* = 0.39
Median duration of MV (hours)	18	24	*P* = 0.32
Median and range PaO_2_ (mmHg) prior to initiation of mechanical ventilation	63; 52–241 (*n* = 10)	90; 40–498 (*n* = 57)	*P* = 0.24
Median and range PCO_2_ (mmHg) prior to initiation of mechanical ventilation	58; 28–130 (*n* = 27)	51; 22–128 (*n* = 124)	*P* = 0.07
Median and range pulse oximetry (%) prior to initiation of mechanical ventilation	88; 76–97 (*n* = 15)	94; 83–100 (*n* = 107)	P = 0.02^*^
Median and range PF ratio prior to initiation of mechanical ventilation	90; 58–482 (*n* = 8)	215; 43–590 (*n* = 124)	P = 0.02^*^
Median and range tidal volume (mL/kg) at initiation of MV	8.5 (4.7–13)	9 (2–25)	*P* = 0.17
Median and range peak inspiratory pressure (cmH_2_O) at initiation of MV	18 (8–37)	15 (5.5-35)	P < 0.01^*^
Median and range peak end expiratory pressure (cmH_2_O) at initiation of MV	8 (2–12)	5 (0–12)	*P* = 0.09
Complications	1 (3.5%)	43 (1.7%)	*P* = 0.08

* *P* < 0.05.

## 4 Discussion

This study revealed no statistical significance between NMBA use and survival rate, MV duration, or complications in dogs undergoing MV, when compared to those who did not receive an NMBA. Neuromuscular blocking agents are utilized in people undergoing MV with mixed conclusions. The proposed advantages of NMBAs include effectively eliminating oxygen consumption and ATP expenditure by respiratory muscles, resulting in decreasing work of breathing (4–6). Respiratory muscle paralysis secondary to NMBA use also increases patient tolerance to hypercapnic acidosis, allowing for provision of low inspiratory tidal volume and low-pressure settings to reduce the risk of ventilator-induced lung injury (8). Additionally, NMBAs may also have direct therapeutic benefits; a multi-center prospective study conducted by Forel et al. determined that NMBA use during MV decreased pneumocyte release of tumor necrosis factor-alpha, interleukin (IL)-1beta, IL-6, and IL-8, which are pro-inflammatory cytokines associated with acute respiratory distress syndrome (5). While beneficial effects of NMBAs exist, there are also disadvantages associated with NMBA use in patients undergoing MV. Neuromuscular blocking agents do not confer any sedating, amnesic, or analgesic properties; therefore, consciousness and pain sensation are preserved when used without adequate sedation/analgesia, which may negatively affect patient outcomes (4, 9, 10). Another safety concern associated with NMBA use in mechanically ventilated patients is ICU-acquired muscle weakness. A study by Segredo et al. revealed that 44% of mechanically ventilated patients who received a continuous infusion of vecuronium, a non-depolarizing NMBA, for at least 48 h experienced prolonged muscular paralysis. These patients were apneic and unable to display spontaneous movement for up to 7 days following discontinuation of the NMBA (11). There are currently no descriptions of ICU acquired weakness in veterinary patients, however, reported complications associated with NMBA administration in veterinary medicine include new onset seizures in 3 mechanically ventilated dogs, hypotension in a dog under general anesthesia for an exploratory laparotomy, and hypersensitivity reactions (12–14).

The double-blinded, randomized, prospective study by Papazian et al., known as the ACURASYS study, determined that the use of NMBAs in patients undergoing MV due to severe ARDS had an improved 90-day mortality rate compared to MV patients who did not receive an NMBA (7). The results of this landmark study, however, conflicts with the findings of the randomized, prospective ROSE study conducted by the National Heart, Lung, and Blood Institute, which revealed no significant difference in 90-day mortality rate between patients with severe ARDS who were administered an NMBA and those who received lighter levels of sedation (15). In the ROSE study, patients in the control group had lighter were able to undergo MV with less sedation and had fewer cardiovascular adverse effects [15]. In a study of children undergoing MV for ARDS, administration of NMBA was associated with increased survival and increased oxygenation index at 48 h (16). The applicability of these studies to veterinary medicine is unclear. Veterinary patients may require higher sedation levels than people to tolerate intubation, regardless of NMBA administration. Neuromuscular blocking agents have been reported in veterinary medicine to facilitate skeletal muscle relaxation during general anesthesia and ocular surgery, however, the description during MV is limited (17).

The overall survival to discharge in this present study was 30.3%, which is similar to previously reported survival to discharge rates of 22% (18). The present study found no significant difference between NMBA administration and survival to discharge. It is additionally important to note that dogs in this study were undergoing MV for a variety of etiologies and the majority of dogs did not meet the diagnostic criteria for severe ARDS. However, dogs that received NMBA agents were significantly more likely to be undergoing MV due to hypoxemia, compared to the group that did not receive NMBAs. Hypoxemia has historically been associated with a lower survival to discharge, compared to hypoventilation.

In this study, dogs in the NMBA group did have significantly lower PF ratios prior to the initiation of MV compared to patients who did not receive an NMBA. This suggests that patients who were administered an NMBA had more severe underlying respiratory disease and impairment in gas-exchange when compared to the non-NMBA group. The similarities in survival rate between the NMBA and non-NMBA patient populations, despite the higher severity of respiratory pathology in the NMBA group, may suggest a potential therapeutic benefit to NMBA use for MV patients. Prospective studies are needed to further explore this relationship. There was no significant difference in use of lung protective ventilation strategies or tidal volume and peak inspiratory pressures at the time of MV initiation in either group. The dogs that received NMBAs had higher PEEP, compared to the non-NMBA group. This may be due to more severe hypoxemia in this group, necessitating use of PEEP at the time of MV initiation.

In addition to the lack of significant impact on survival to discharge, there was no significant difference on the incidence of complications, including pneumothorax, between dogs that did and did not receive NMBAs. It is proposed that NMBAs may reduce the incidence of barotrauma and resultant pneumothorax by eliminating patient-ventilator dyssynchrony. Breath stacking, a form of dyssynchrony that occurs when the ventilator delivers a breath before a patient fully expires, results in progressive increases in tidal volume. Administration of an NMBA effectively eliminates breath stacking, thereby reducing intrathoracic pressure and the risk of barotraumatic lung injury (19). The lack of difference in this study may be explained by the relatively low incidence of pneumothorax.

Patients who receive an NMBA experience global muscle paralysis and are unable to swallow, gag, or otherwise protect their airways, however, use of NMBA in this study was not associated with a higher incidence of ventilator associated pneumonia. It is important to note that the median duration of MV in the NMBA group was only 18 h. We suspect that a relatively small sample size in the NMBA group contributed to this result. However, additional studies investigating the relationship between NMBA use and the risk of VAP are warranted.

Other complications reported to be associated with NMBA administration in dogs include new onset seizures and allergic reactions, neither of which were noted in this population of patients (12, 14). Other possible complications including hypotension were not evaluated in this study due to the multitude of factors associated with hypotension in this patient population.

There were limitations to this study. The retrospective nature of this study did not allow for control of multiple variables, including ventilator settings, nursing and supportive care measures, and the assignment of study groups. The reason for NMBA administration, including facilitation of sedation, or management of dyssynchrony was not available. All dogs undergoing MV were included in this study, regardless of primary etiology. Therefore, dogs undergoing MV for lower motor neuron disease were included in both groups and may have affected results due to the potential paralysis associated with lower motor neuron disease.

The sample size for the NMBA group was relatively small, which decreased the statistical power of our study and the efficacy of NMBAs was not assessed. While the multicenter nature of the study allowed for the inclusion of a large number of patients, treatment differences between locations may have resulted in numerous variables that may have impacted patient outcomes.

In conclusion, our study revealed no statistical significance between NMBA use and survival rate in dogs undergoing MV; however, dogs that received NMBA did have evidence of worse pulmonary function, which may indicate that NMBA may be beneficial in severely hypoxemic patients. Further investigation into the advantages and limitations of NMBA use in dogs undergoing mechanical ventilation may aid in our ability to support this critically ill patient population and improve patient outcomes.

## Data Availability

The raw data supporting the conclusions of this article will be made available by the authors, without undue reservation.
